# Exploring Patient Loyalty: The Effect of Entrepreneurial Leadership Through Corporate Governance, Service Quality, and Satisfaction

**DOI:** 10.12688/f1000research.166837.1

**Published:** 2025-10-03

**Authors:** Iing Ichsan Hanafi, Taher Alhabsji, Kadarisman Hidayat, Ari Darmawan

**Affiliations:** 1 Business Administration, Universitas Brawijaya, Malang, East Java, 65145, Indonesia

**Keywords:** entrepreneurial leadership, corporate governance, service quality, patient satisfaction, sustainable patient loyalty, healthcare management

## Abstract

**Background:**

It is imperative to deliver exceptional services in order to improve patient contentment and cultivate enduring loyalty in the healthcare sector, which is becoming more competitive. The direct impact of leadership and governance on patient experience and retention remains underexplored, despite the increasing recognition of their role in determining care delivery. This investigation investigates the connections between healthcare organizations’ corporate governance, service quality, patient satisfaction, and loyalty, as well as entrepreneurial leadership.

**Methods:**

Data were obtained from 384 executive patients in hospitals throughout Indonesia using structural equation modeling (SEM).

**Results:**

The findings indicate that corporate governance and service quality are considerably improved by entrepreneurial leadership, while patient satisfaction and loyalty are positively impacted by corporate governance. Nevertheless, patient satisfaction was found to be moderately influenced by service quality, while patient loyalty was significantly influenced. As a result, patient satisfaction was demonstrated to be a critical factor in the development of patient loyalty. These results emphasize the importance of high service quality, effective governance, and entrepreneurial leadership in promoting patient satisfaction and loyalty.

**Conclusions:**

The research offers valuable insights for healthcare managers and policymakers who are interested in enhancing organizational performance and improving patient retention. This research theoretically enhances comprehension of the impact of leadership and governance frameworks on patient retention and service quality. In practical terms, it provides valuable recommendations for hospital administrators and policymakers to improve patient satisfaction and loyalty by implementing effective leadership, robust governance, and continuous service improvement.

## 1. Introduction

The loyalty of patients is becoming an increasingly important factor in determining an organization’s ability to grow and develop over the long term in the healthcare industry. The ability to retain patients not only ensures a steady revenue stream but also contributes to a healthcare organization’s resilience in the face of fluctuating market conditions and changing healthcare demands ( Senyapar, 2024; Zhang et al., 2018). However, achieving and maintaining patient loyalty requires much more than just providing medical care. It necessitates a holistic understanding of the factors that influence patients’ decision to remain with a healthcare provider over time, which are influenced by their experiences, perceptions, and emotional connections formed within the healthcare setting ( Chunmei & Zhang, 2018). As such, loyalty in healthcare is becoming increasingly recognized as a multifaceted construct that extends beyond transactional exchanges between patients and providers. It is deeply interwoven with factors such as service quality, patient satisfaction, and the broader ethical dimensions of care delivery ( Monferrer et al., 2019; Nguyen et al., 2021; Singh & Dixit, 2020). This understanding has led to a shift in focus, from merely delivering care to cultivating lasting, meaningful relationships with patients.

Healthcare providers must be perceived as competent, trustworthy, and adaptable to the changing needs and expectations of patients. This is especially crucial in the context of the increasing prevalence of digital health instruments that increase patient empowerment and their participation in healthcare decision-making ( Clark et al., 2006). In spite of these developments, numerous healthcare organizations in Indonesia encounter difficulties in maintaining patient loyalty. This is frequently the result of inconsistent service quality, inadequate governance structures, and the inability to adjust to new technological trends ( Hsieh & Lin, 2022).

**Figure 1.**
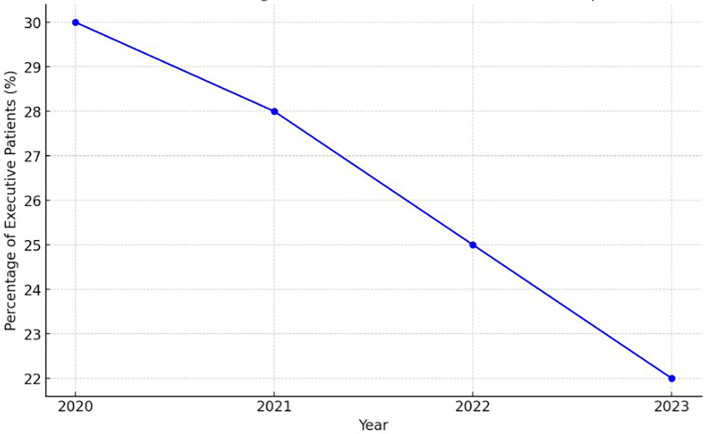
Estimated decline in percentage of executive patients in Indonesian hospitals (2020-2023). Source: Internal Report of Indonesian Hospitals (2023).

The decline in executive patients, who contribute significantly to the revenue from outpatient and inpatient services, has become a pressing issue for many hospitals in Indonesia. Data from 2020 to 2023 show an average annual decrease of 15% in the percentage of executive patients in Figure 1, which directly impacts the hospitals’ financial stability and operational efficiency. This trend raises important questions about the underlying causes of the decline and the strategic efforts required to address the situation.

One major factor contributing to this decline is the growing patient expectation for service quality, largely driven by digital health tools and better access to information. As healthcare professionals strive to satisfy these heightened expectations, patients are more inclined to transfer doctors if their needs are not addressed ( Berry, 2019). Issues such as communication gaps, long waiting times, and limited accessibility to healthcare services further contribute to patient dissatisfaction, weakening patient trust and engagement over time ( Batbaatar et al., 2017; Siddique et al., 2024).

Patients now have more options than they ever have before, which means that healthcare institutions are having to contend with fierce competition ( Berry, 2019). To remain competitive and continue to provide value, it is crucial for healthcare organizations to leverage strategies that ensure the loyalty of their patients ( Rivers & Glover, 2008; Senyapar, 2024). In addition to being more likely to use the facility again in the future, loyal patients are also more inclined to refer others to it, acting as representatives of the company’s reputation and core principles ( George & Sahadevan, 2019; Poku et al., 2017). This aspect of patient loyalty, particularly in an environment where patient choice is at an all-time high, underscores its importance as a strategic asset for healthcare institutions. While traditional approaches focus on quality of care and patient treatment outcomes, patient loyalty encompasses much more. It depends on elements that are thought to be essential to establishing and preserving long-term relationships with patients, such as emotional fulfillment, the patient experience, and the provision of individualized care ( Iversen et al., 2025; Li et al., 2024). It is crucial to have a thorough awareness of the elements that promote patient loyalty within this framework. An organization in the healthcare industry that is able to successfully negotiate these difficulties is in a position to maximize its competitive advantage and maintain its current level of performance.

The understanding that leadership has a significant impact on healthcare results is at the heart of our investigation on patient loyalty ( Cummings et al., 2008; Patri & Suresh, 2018; Strömgren et al., 2017). Specifically, in the healthcare industry, patient loyalty is mostly dependent on innovation, adaptability, and patient-centered approaches, all of which are fostered by entrepreneurial leadership ( Sayyed et al., 2025). Entrepreneurial leadership, distinct from traditional management models, is characterized by a forward-thinking, proactive approach to leadership, emphasizing innovation, risk-taking, and the ability to address challenges in novel ways ( Awad et al., 2024; Borde et al., 2022). This leadership style enables healthcare organizations to adapt to shifting demands, technological advancements, and evolving patient expectations in ways that enhance patient satisfaction and, by extension, loyalty ( Adiguzel, 2025; Raderstorf, 2024).

Entrepreneurial leaders are adept at recognizing opportunities for improvement and driving change within organizations ( Koryak et al., 2015; Wu et al., 2025). The entire patient experience and patient outcomes can be directly impacted by the implementation of novel technologies, the introduction of new models of care, or the improvement of operational efficiency. Entrepreneurial leaders’ ability to establish a culture of continuous improvement and foster an environment that encourages risk-taking and problem-solving is crucial for adapting to the rapidly evolving healthcare landscape ( Mehmood et al., 2021; Wu et al., 2025). For instance, by leveraging the latest medical technologies and adopting patient-centric strategies, entrepreneurial leaders can improve the quality of care and make patients feel valued, ultimately reinforcing their loyalty to the institution. This approach contrasts with traditional models of leadership that may prioritize administrative efficiency or hierarchical decision-making. Entrepreneurial leadership places greater emphasis on empowering employees, fostering innovation, and encouraging collaboration at all levels of the organization ( Mehmood et al., 2021; Newman et al., 2018). This empowerment is crucial in ensuring that the staff feels motivated to contribute to improving the patient experience, whether through direct care or support roles.

Corporate governance is also very important in the healthcare business since it helps entrepreneurial executives carry out their plans. Effective corporate governance practices help ensure that healthcare organizations operate transparently and ethically, which, in turn, enhances patient trust and satisfaction ( Sayyed et al., 2025). Corporate governance frameworks define the processes, structures, and policies that guide the organization’s operations, providing oversight and accountability for decisions that affect patient care ( Barbazza & Tello, 2014). Maintaining good governance standards allows healthcare businesses to manage risks, assure regulatory compliance, and develop an ethical decision-making culture, all of which contribute to patient trust. As patient loyalty is closely tied to perceptions of trust, effective corporate governance becomes a fundamental mechanism for securing patient confidence and long-term loyalty.

Service quality is another fundamental determinant of patient loyalty, refers to the overall effectiveness of healthcare services in meeting or exceeding patients’ expectations ( Chehayeb, 2023). The quality of service is strongly linked to patient satisfaction and is a fundamental component in fostering patient loyalty ( Alodhialah et al., 2024; Coutinho et al., 2019; Divya et al., 2025; Fatima et al., 2018; Monferrer et al., 2019; Olesen & Bathula, 2022; Özden & Çelik, 2025). In a healthcare setting, service quality encompasses not just clinical outcomes but also the emotional and psychological aspects of patient care ( Cadet & Sainfort, 2023; Endeshaw, 2021). Factors such as the demeanor of healthcare staff, the clarity of communication, the responsiveness of the organization to patient needs, and the overall comfort and convenience of the healthcare environment play significant roles in shaping the patient experience. When patients perceive high service quality, they are more likely to feel valued and respected, which strengthens their emotional connection to the healthcare provider and encourages repeat visits ( Dinur et al., 2025).

Additionally, loyalty in healthcare continues to be driven by satisfaction ( Liu et al., 2021; Meesala & Paul, 2018; Rather & Hollebeek, 2019). Baker et al. (2021) and Tan et al. (2023) have both identified patient satisfaction as an emotional outcome that is influenced by the patient’s evaluation of the care they receive. This evaluation is influenced by factors such as the interaction with healthcare professionals, the perceived competence of the medical staff, and the overall atmosphere of the healthcare environment. The institution’s reputation and brand image can be significantly impacted by patients who are satisfied with their care, as they are more likely to return for additional services and to disseminate positive word of mouth ( Fattahi et al., 2022; Dandis et al., 2022). Consequently, the connections between corporate governance, service quality, satisfaction, and loyalty, as well as entrepreneurial leadership, are intricate yet profoundly interconnected.

Overall, the multifaceted nature of patient loyalty in healthcare necessitates a comprehensive and integrated approach to understanding its drivers. Entrepreneurial leadership, corporate governance, service quality, and patient satisfaction all play crucial roles in fostering and sustaining loyalty. Healthcare businesses can deal with the problems of today’s healthcare system, come up with new ideas, and make sure that patients get the best care possible by embracing entrepreneurial leadership. By doing this, they build a foundation for long-term patient loyalty, which is important for their long-term success and ability to compete in the healthcare field.

## 2. Literature review and hypothesis development

### 2.1 The relationship between entrepreneurial leadership and corporate governance

Innovative ideas, being proactive, taking risks, and being able to see and take advantage of new chances are all important parts of entrepreneurial leadership ( Clarks et al., 2019; Kebede et al., 2024). Achieving long-term success in the fast-paced corporate world of today requires leaders to be flexible and have an entrepreneurial mindset. This type of leadership goes beyond traditional management practices by fostering a culture of creativity, flexibility, and continuous improvement, all of which are crucial in driving organizational performance.

On the other hand, corporate governance encompasses the systems, structures, and procedures that guarantee that organizations are effectively managed and operated in compliance with ethical standards, laws, and regulations ( Shleifer & Vishny, 1997). Strong corporate governance practices are vital for establishing transparency, accountability, and trust within organizations, which are necessary for sustainable growth and stakeholder confidence.

Entrepreneurial leadership has a significant influence on corporate governance by encouraging innovative governance structures and practices. Entrepreneurial leaders are more likely to prioritize transparency, ethical decision-making, and accountability, as they recognize the long-term value these practices bring to the organization. They tend to foster open communication and a decentralized decision-making structure, which enhances the responsiveness and adaptability of the organization. In addition, entrepreneurial leaders are proactive in anticipating challenges and opportunities, and they tend to implement governance practices that enable organizations to remain competitive and agile in the face of change ( Renko et al., 2015; Awad et al., 2024).

Moreover, entrepreneurial leadership promotes a culture of risk management and responsibility. Leaders with an entrepreneurial orientation understand the importance of managing risks while pursuing opportunities, ensuring that corporate governance practices are designed to mitigate potential risks and enhance organizational stability. Additionally, they are more inclined to advocate for ethical governance and sustainability, underscoring the significance of long-term value generation over immediate benefits. Given these considerations, it is hypothesized that:

H1. Entrepreneurial leadership has a significant effect on corporate governance.

### 2.2 The relationship between entrepreneurial leadership and service quality

In contemporary organizations, entrepreneurial leadership has become a critical factor in determining success, particularly in service-oriented industries. This leadership style blends traditional leadership competencies with entrepreneurial thinking, allowing leaders to foster innovation, adaptability, and continuous improvement ( Clarks et al., 2019; Kebede et al., 2024). Unlike conventional leadership models, which may focus more on efficiency and stability, entrepreneurial leadership emphasizes proactivity, risk-taking, and the identification of opportunities, all of which are crucial in dynamic service environments ( Renko et al., 2015). Leaders with entrepreneurial orientation encourage creativity, empower employees to propose innovative solutions, and emphasize the importance of customer-centric strategies, which are fundamental to achieving high service quality ( Al-Dhaafri et al., 2016; Kim et al., 2018).

Service quality, in turn, is a critical factor influencing customer satisfaction and loyalty. In service industries, where the interaction between employees and customers plays a central role, delivering consistent, high-quality service is paramount. Entrepreneurial leadership promotes a culture that nurtures continuous improvement, responsiveness, and active customer engagement, all of which directly contribute to enhancing service quality ( Hoang et al., 2024; Kebede et al., 2024). By encouraging innovation and creativity, entrepreneurial leaders help organizations respond to the evolving demands of customers, which is essential in maintaining a competitive edge in the marketplace.

Entrepreneurial leaders motivate their teams to innovate and take calculated risks, fostering a proactive mindset that is crucial for identifying new opportunities to improve service delivery ( Corrêa et al., 2022). Such leadership is particularly valuable in environments where service quality is not solely defined by standardized procedures but also by the ability to adapt to customer needs and expectations. The capacity of entrepreneurial leaders to establish an environment that encourages risk-taking and experimentation is crucial for organizations to enhance their service offerings and uphold a high level of quality, which is crucial for customer retention and the development of long-term loyalty.

Furthermore, entrepreneurial leadership’s emphasis on customer engagement and relationship-building directly impacts service quality. Leaders with an entrepreneurial orientation understand the importance of aligning organizational goals with customer needs, ensuring that service delivery is not just efficient but also tailored to the customer experience ( Renko et al., 2015). This approach leads to the development of services that are not only effective but also resonate with customers on an emotional level, fostering stronger customer relationships and satisfaction. Through these endeavors, entrepreneurial leadership establishes a strong foundation for ongoing enhancement, which subsequently enhances organizational performance and service quality. Given these insights, the following hypothesis is proposed:

H2. Entrepreneurial leadership has a significant effect on service quality.

### 2.3 The relationship between corporate governance and patient satisfaction

An organization’s overall performance and success are greatly influenced by corporate governance, especially in service-oriented industries like healthcare. It encompasses the structures, processes, and policies that guide organizational decision-making, ensuring transparency, accountability, and ethical practices ( Al Astal et al., 2024; Aguilera & Ruiz Castillo, 2025). In healthcare, strong corporate governance is essential for maintaining high standards of service delivery, ensuring that the interests of patients are prioritized and that healthcare organizations operate in a responsible and sustainable manner ( Shleifer & Vishny, 1997).

In turn, patient satisfaction is a critical outcome of the delivery of healthcare services. The patient’s evaluation of the care they received is encapsulated in the statement, which is influenced by the comprehensive patient experience, the availability of resources, the proficiency of healthcare professionals, and the quality of medical treatment. The organization’s long-term success and reputation are directly impacted by the probability of satisfied patients returning for future treatments, recommending the healthcare provider to others, and engaging in positive word-of-mouth ( Huang et al., 2021).

Corporate governance influences patient satisfaction by ensuring that healthcare organizations operate with integrity, transparency, and a commitment to continuous improvement. Organizations with strong governance structures are more likely to prioritize patient-centered care, invest in staff training, and implement policies that enhance the overall patient experience. Effective governance ensures that patient safety, privacy, and quality of care are always top priorities, which are crucial for building trust with patients.

Moreover, governance structures that promote accountability and ethical decision-making foster an environment where healthcare providers are incentivized to meet or exceed patient expectations ( Knowles, 2024). When governance mechanisms are in place to monitor performance, allocate resources effectively, and maintain ethical standards, patients are more likely to perceive the healthcare institution as reliable and trustworthy, which significantly enhances their satisfaction ( Meesala & Paul, 2018; Sadeh, 2017). Given these considerations, it is hypothesized that:

H3. Corporate governance has a significant effect on patient satisfaction.

### 2.4 The Relationship Between Corporate Governance and Patient Loyalty

The long-term success and sustainability of healthcare organizations are significantly influenced by corporate governance. It pertains to the organizational systems, structures, and procedures that guarantee accountability, transparency, and ethical decision-making, all of which are indispensable for the organization’s efficient operation ( Shleifer & Vishny, 1997). In healthcare, corporate governance involves policies and practices that prioritize patient care, safety, and satisfaction, while also ensuring compliance with legal and ethical standards. Strong governance frameworks are necessary for fostering trust and confidence among patients, which directly influences their loyalty to healthcare providers.

Patient loyalty is the term used to describe a patient’s enduring dedication to a specific healthcare provider, which is influenced by their contentment with the services they receive, trust, and positive experiences. The healthcare organization’s reputation, growth, and financial stability are substantially influenced by the likelihood of loyal patients returning for future medical care, adhering to prescribed treatment plans, and recommending the provider to others. Building patient loyalty necessitates not only the provision of high-quality care, but also the organization’s adherence to ethical standards, transparency, and responsiveness to patient needs and concerns.

Corporate governance influences patient loyalty by creating an environment of trust and reliability. Healthcare organizations with strong governance practices are more likely to implement policies and procedures that align with patient needs, ensuring that services are consistently high-quality, accessible, and patient-centered. Effective governance fosters transparency in decision-making, ensures the proper allocation of resources, and promotes ethical practices that prioritize patient well-being. When patients perceive that a healthcare organization operates with integrity and accountability, they are more likely to develop a strong emotional attachment to the provider, which is essential for fostering long-term loyalty ( Meesala & Paul, 2018).

Moreover, strong corporate governance encourages healthcare organizations to be responsive to patient feedback and continuously improve the quality of care. By adopting patient-centered policies, maintaining high standards of care, and ensuring that patients’ concerns are addressed in a timely and empathetic manner, organizations build a loyal patient base. Ethical governance practices, such as protecting patient privacy, enhancing the patient experience, and ensuring transparency in billing and treatment options, are crucial for building trust and loyalty. Patients who trust their healthcare provider are more likely to remain loyal, even in the face of alternative options ( Sadeh, 2017; Malenfant et al., 2022). Given these considerations, it is hypothesized that:

H4. Corporate governance has a significant effect on patient loyalty.

### 2.5 The Relationship Between Service Quality and Patient Satisfaction

In healthcare contexts, service quality is a critical factor in determining patient satisfaction, as it directly impacts patients’ perceptions of value, trust, and their overall experience with a healthcare provider ( Akthar et al., 2023; Alfarizi, 2022). The notion of service quality incorporates a variety of dimensions, such as the tangible aspects of care that meet or exceed patient expectations, reliability, responsiveness, assurance, and empathy ( Parasuraman et al., 1988; Zeithaml et al., 1996). Patients are more likely to have positive feelings about a hospital if it regularly provides high-quality services. This leads to higher levels of happiness and greater loyalty ( Fatima et al., 2018; Kim et al., 2017; Shabbir et al., 2016). In contrast, bad service quality, like treatment delays, staff members acting rudely, or not meeting patients’ needs, can cause dissatisfaction, bad word-of-mouth, and patients leaving, which hurts healthcare institutions’ reputation and bottom line ( Agarwal et al., 2024; Gangopadhyay & Ukil, 2022).

Patients evaluate their experiences not only based on clinical outcomes but also on factors such as the attentiveness of healthcare providers, the clarity of communication, the cleanliness of facilities, and the ease of accessing services. Patients feel better about their care when the service is of high quality, which builds trust and emotional connections with healthcare workers. This is very important in the healthcare field, where patients’ mental and emotional health is just as important as their physical health ( Fatima et al., 2018; Meesala & Paul, 2018). Patients who are happy are not only more likely to return for more care, but they are also more likely to tell others about the healthcare center, which improves its reputation and helps it succeed in the long run ( Moreira & Silva, 2015; Smith, 2021).

Oliver’s (1980) Expectation Disconfirmation Theory is a useful way to think about how service quality affects customer satisfaction. This idea says that happiness happens when what the patient expected and what they actually got don’t match up. When the quality of the service goes above and beyond what the patient expected, it leads to positive disconfirmation and high levels of happiness. If the quality of the service doesn’t live up to standards, on the other hand, negative disconfirmation happens, which makes the customer unhappy. This theory shows how important it is to meet and go beyond what patients expect to make sure they are completely satisfied. When it comes to healthcare, this means that people who get consistent, reliable, and quick service are more likely to feel like their needs were met or exceeded, which makes them happier.

Patients are also rendered pleased by the quality of their service, which cultivates emotional connections and confidence between them and their healthcare providers ( Fatima et al., 2018; Meesala & Paul, 2018; Shan et al., 2016). A patient is more inclined to believe that the care they receive is of high quality if they have faith in their healthcare provider, even in the face of obstacles or complications during treatment. Patients who have a positive emotional experience and trust their therapists are more likely to be satisfied with the service as a whole. This increases the probability of their return and the establishment of enduring relationships. Service quality also enhances patient satisfaction by ensuring that patients feel valued and respected throughout their healthcare journey, from the initial contact to the follow-up after treatment.

Hospitals that prioritize continuous service improvements—whether through staff training, adopting the latest technologies, or implementing patient feedback mechanisms—are better equipped to enhance patient satisfaction ( Bhati et al., 2023; Lee et al., 2012; Sunder et al., 2020). By fostering an environment of continuous improvement, healthcare institutions demonstrate their commitment to delivering high-quality care that evolves in response to patient needs and expectations. Training staff to be responsive, empathetic, and attentive to patient concerns is essential in improving the quality of service, as these interpersonal factors significantly influence patient satisfaction. Moreover, hospitals that actively seek and act on patient feedback can identify areas for improvement and ensure that the services provided align more closely with patient needs.

Service quality affects more than just happiness. It also affects brand loyalty and patient retention, which are very important for the long-term success of healthcare organizations ( Meesala & Paul, 2018; Shabbir et al., 2016; Vimla & Taneja, 2021). When patients are happy with the quality of care they receive, they are more likely to return for more care and tell their family and friends about the provider. There are more patients who stay with the practice, and their group of loyal patients grows. Healthcare companies that put a lot of emphasis on service quality are better able to build and keep long-term relationships with their customers. This makes them more competitive in the market. Thus, the following hypothesis is proposed:

H5. Service quality has a significant effect on patient satisfaction.

### 2.6 The relationship between service quality and patient loyalty

Service quality is one of the most important factors in keeping patients coming back, which is important for healthcare workers’ long-term success and survival. Patients’ trust, satisfaction, and desire to build and keep long-term relationships with their healthcare providers are all directly affected by the quality of the care they receive ( Parasuraman et al., 1988). Service quality characteristics like dependability, responsiveness, empathy, and competence are important for giving patients good experiences that make them more loyal to a certain provider ( Cadet & Sainfort, 2023; Chen et al., 2024; Hannawa et al., 2022). When patients regularly feel these things about their healthcare provider, they are more likely to form a strong bond with them, which is important for keeping patients loyal. On the other hand, patients are more likely to switch providers if they think the level of service is poor or inconsistent. This makes it harder to keep patients and keep them loyal.

The Social Exchange Theory ( Naidu, 2009; Swain & Kar, 2018) is a useful way to look at the connection between good service and loyal customers. This idea says that people interact with each other because they expect to get something in return. People who need medical care want value in the form of high-quality care, individualized treatment, and caring interactions with medical experts. People who have these needs met feel like the healthcare provider values their time, trust, and resources, which makes them more loyal to that provider. Patients who feel valued, respected, and cared for are more likely to return for future visits and tell others about the provider. This helps the provider get more patients and improves its image ( Malenfant et al., 2022; Sinclair et al., 2016).

Service quality and patient loyalty are linked in a good way, and emotional ties and trust make that link even stronger. Patients feel safe and confident in their healthcare provider when they consistently receive high-quality care. This makes them less likely to look for care elsewhere ( Alodhialah et al., 2024; Widiastuti et al., 2024). These emotional bonds and trust are crucial for the long-term retention of patients. Trust, in particular, is a key determinant of patient loyalty, as it influences patients’ decisions to stay with a provider and seek ongoing care for future health needs. A healthcare provider that consistently demonstrates reliability, expertise, and genuine concern for patients’ well-being fosters a deeper emotional connection with patients, reinforcing their loyalty over time.

Service quality also plays a critical role in patient engagement, another crucial factor in sustainable loyalty. Healthcare providers that continuously focus on improving service delivery, ensuring patient-centered care, and engaging in transparent communication are more likely to retain their patients and encourage long-term relationships ( Fukami, 2024; Omaghomi et al., 2024). Such institutions create an environment in which patients feel heard, valued, and understood, thus enhancing their overall experience. This emphasis on continuous service improvement and patient satisfaction not only leads to immediate patient retention but also lays the foundation for enduring loyalty. Additionally, healthcare providers who are transparent in their communications and address patient concerns in a timely and empathetic manner are more likely to foster positive relationships that transcend one-time visits and encourage ongoing care.

Moreover, healthcare organizations that prioritize service quality are better positioned to create a differentiated competitive advantage. In an increasingly competitive healthcare environment, where patients have more choices than ever, organizations that provide superior service quality stand out. The consistency with which a provider delivers high-quality care influences patients’ perceptions of the provider’s commitment to their well-being, thus strengthening the emotional connection between patients and the institution. This emotional connection is crucial for long-term loyalty and retention. Patients who feel that their healthcare provider genuinely cares for them are more likely to remain loyal, even in the face of external options that may appear more convenient or less costly.

Service quality affects patient loyalty in more ways than just clinical results. Patients’ general loyalty to a healthcare provider is based on how happy they are with their interactions with staff, how easy it is to get services, and how nice the space is. Patients are more likely to stay with the same healthcare provider for a long time if they think their whole experience, from making appointments to following up after care, was excellent. This all-around view of service quality makes sure that patient loyalty isn’t just based on medical results, but also on the whole service experience that healthcare institutions offer. Thus, we hypothesize:

H6. Service quality has a significant effect on patient loyalty.

### 2.7 The relationship between patient satisfaction and patient loyalty

Long-term patient loyalty in healthcare depends a lot on how happy and satisfied the patient is. An established link has been found between these two factors; happy patients are more likely to stay loyal to their healthcare providers, return for more care, and suggest the provider to others ( Meesala & Paul, 2018). Patient satisfaction has an effect on loyalty because of both the good experiences that happen right away and the emotional ties and trust that are built over time. When it comes to healthcare, satisfaction can mean a lot of different things, such as the quality of communication, responsiveness, dependability, empathy, and the general service experience. When these things match up with what the patient wants, they make an emotional link that leads to long-term loyalty.

A good way to understand how patient happiness affects loyalty is to look at social exchange theory. This idea says that ties between people and groups are based on exchanges that are good for both sides ( Naidu, 2009; Swain & Kar, 2018). The exchange in healthcare means giving people high-quality care and services in exchange for their time, trust, and money. Patients are more likely to keep working with the same provider if they think they got good value for their time through quick, responsive care and a positive overall experience. This exchange of value makes the relationship between the patient and the healthcare provider stronger, which makes the patient happier and, in the end, more loyal.

Researchers have found that patients who are happy with their care are more likely to form strong emotional bonds with their doctors, which can lead to long-term trust ( Dayan et al., 2022; Kumah, 2019; Vink et al., 2019). These patients believe their providers and feel safe with their care, which makes them less likely to look for services from other companies. This emotional connection goes beyond just getting what you want; it turns into a commitment to the healthcare worker as a person. Not only are happy patients likely to return for future medical needs, they also act as champions for the provider, telling family and friends about the healthcare institution, which makes them even more loyal ( Sadeh, 2017; Zhang et al., 2022). The main idea behind this is that patient happiness both leads to loyalty and helps keep that loyalty strong over time.

Additionally, patient satisfaction plays a crucial role in reducing patient churn, which is the process of patients discontinuing services or switching providers. In highly competitive healthcare environments, where patients have a wide range of options, dissatisfaction can quickly lead to patient attrition. However, when patients feel satisfied with their care, they are less likely to consider alternatives. High satisfaction levels also contribute to patient retention, which is particularly valuable in healthcare settings where long-term relationships with patients are crucial for sustained organizational success. When healthcare providers consistently meet or exceed patient expectations, they reduce the likelihood of patient churn and build a loyal patient base ( Huang et al., 2021; Liu et al., 2021).

Also, the emotional and relational parts of caring for a patient are just as important for building long-term loyalty as the clinical results. The emotional and mental needs of patients are closely linked to how they see the level of care, which in turn affects how satisfied they are. For instance, patients who feel like their healthcare providers listen, understand, and value them are more likely to be very satisfied, which makes them more loyal to that provider. This makes a point of showing that patient happiness isn’t just about how well they did in the medical setting, but also about how well they were treated by other people. When healthcare providers focus on these aspects of care, customer satisfaction goes up, which leads to more loyal patients. Based on these considerations, the following hypothesis is proposed:

H7. Patient satisfaction has a significant effect on patient loyalty.

**Figure 2.**
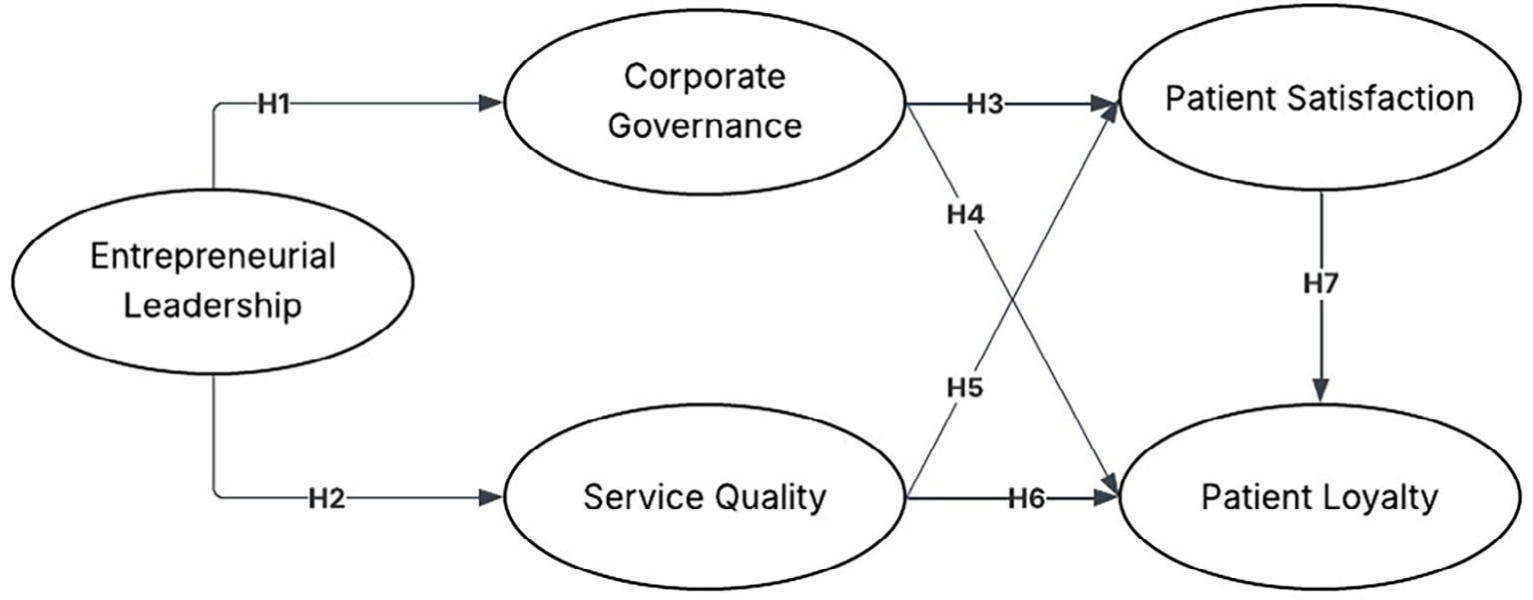
Hypothesis model. Source: Author’s Own Work (2025).

Based on this explanation, the hypothesis model can be seen in Figure 2.

## 3. Research methods

### 3.1 Population and sample

The population for this study consists of executive patients who have received medical treatment at hospitals located in West Java, Indonesia. This regional focus ensures that the research reflects the specific healthcare environment within this area. A total of eight hospitals were selected from a pool of 50 hospitals in West Java, based on the following criteria: each hospital must have more than 200 beds and be classified as Type B. Type B hospitals were chosen due to their capacity to provide comprehensive healthcare services and their significant patient volume, making them representative of the broader healthcare system in the region.

The total population of executive patients across these hospitals is 9,030. The inclusion criteria for this study were executive patients who have visited the hospital at least twice within the past year. A sample size of 384 was calculated using the Slovin formula with a 5% margin of error, ensuring that the sample size was statistically valid for the given population. The formula used is:

$$n=\frac{N}{1+Ne^{2}}=\frac{9030}{1+9030*0.5^{2}}=383,03\approx 384$$

Using this formula, the total sample size of 384 was determined. To ensure proportional representation across the hospitals, the sample was then distributed proportionally based on the total number of patients at each hospital. To calculate the proportional sample for each hospital, the following equation was used:

$$\text{Proportional Sample}=\frac{\text{Total Patient}\;\mathrm{at}\;\text{Hospital}}{\text{Total Population}}\times \;\text{Total Sample Size}$$

The total number of patients and the proportional sample across the selected hospitals is shown in Table 1.

**Table 1.** Population of selected hospitals in West Java, Indonesia.

No	Hospital location	Total patients	Sample size
1	Jatinegara	1,213	52
2	Kemayoran	890	38
3	Bekasi	2,047	87
4	Depok	1,275	54
5	Daan Mogot	1,219	52
6	Bogor	1,030	44
7	Pasteur	919	39
8	Grand Wisata	437	19
Total		9,030	384

Source: Author’s processing (2025).

Ethical approval for this study was obtained from Universitas Brawijaya, effective from September 2024. The ethical approval process ensured patient confidentiality, informed consent, and that no coercion was involved in participation. This study was conducted from September to October 2024, with ethical approval obtained prior to the commencement of data collection.

### 3.2 Variables measurement

A structured questionnaire made to measure key variables linked to the study was used to collect the data for this study. The way each variable was measured was based on well-known categories and scales from other research. Items from questionnaires that were used in other studies were changed to fit the needs of this study better. To find out how much people agreed with each statement, a 5-point Likert scale was used for each variable. The scale went from “strongly disagree” to “strongly agree.”

Entrepreneurial Leadership ( Abubakar et al., 2018) was measured by assessing statements that reflect the leader’s role in fostering innovation, creating solutions for complex problems, being proactive in facing challenges, and having a clear strategic vision. The specific statements included: “The leader encourages innovation in the organization,” “The leader creates creative solutions for complex problems,” “The leader is proactive in facing challenges,” and “The leader has a clear strategic vision.”

Corporate Governance ( Novatiani et al., 2022) was measured through statements that reflect transparency, accountability, and social responsibility. The relevant statements included: “Important information is communicated openly to stakeholders,” “The company is accountable for every decision it makes,” and “The company is actively involved in social responsibility.”

Service Quality ( Altuntas et al., 2012) was assessed using statements based on the SERVQUAL model, which includes dimensions like reliability, responsiveness, assurance, and empathy. The statements used to measure service quality were: “The service provided meets expectations,” “The staff shows empathy toward patients,” “The medical staff understands the emotional needs of patients,” “The staff responds quickly to the needs of patients,” and “The staff possesses good competency in delivering services.”

Patient Satisfaction ( Abekah-Nkrumah et al., 2021) was measured by statements reflecting whether patient expectations were met, their comfort during service delivery, and their emotional connection to the healthcare provider. The statements included: “The patient’s expectations of the service are met,” “The patient feels comfortable during the service process,” and “The patient has an emotional bond with the hospital.”

Patient Loyalty ( Abekah-Nkrumah et al., 2021) was measured by statements evaluating the patient’s intention to return, recommend the service, and trust the healthcare provider. The relevant statements were: “The patient intends to return to use the service,” “The patient will recommend the service to others,” and “The patient has full trust in the hospital’s services.”

### 3.3 Data analysis techniques

The data were analyzed with Partial Least Squares (PLS) via SmartPLS 4.0. PLS is a resilient technique that does not necessitate the data to conform to the normalcy assumptions of conventional statistical tests. This study is well-suited for examining correlations among various variables and forecasting their effects. The investigation sought to assess the interconnections among entrepreneurial leadership, corporate governance, service quality, satisfaction, and patient loyalty.

To mitigate any common method bias, the study meticulously crafted the questionnaire items to reduce response biases, specifically by eliminating leading or confusing questions. The measurement devices’ validity and reliability were rigorously evaluated. Construct validity was evaluated by correlation analysis to confirm that the items sufficiently represented the target constructs. Reliability was assessed using Cronbach’s alpha, with all constructs surpassing the conventional criterion of 0.70, signifying strong internal consistency.

## 4. Results

### 4.1 Demographic respondent

Table 2 provides a summary of the demographic characteristics of the respondents. The following important characteristics are present in the sample of 384 respondents:

**Table 2.** Demographic information of respondents.

Characteristic	Frequency	Percent (%)
**Gender**		
Male	287	74.7
Female	97	25.3
**Education level**		
High school	136	35.42
Diploma	96	25
Bachelor’s degree	77	20.05
Master’s degree	66	17.19
Doctorate	9	2.34
**Age group**		
0-17 years	8	2.08
17-30 years	99	25.78
30-40 years	148	38.54
40-50 years	82	21.35
50-60 years	38	9.90
Above 60 years	9	2.34
**Occupation**		
Private sector employee	195	50.78
Housewife	73	19.01
Entrepreneur/self-employed	35	9.11
Government employee/Army/Police	20	5.21
Other	61	15.89

Source: Author’s processing (2025).

Table 2 illustrates the demographic attributes of the respondents, indicating a heterogeneous sample regarding gender, education, age, and occupation. The bulk of responders were male, constituting 74.7% (287), and females accounted for 25.3% (97). The majority of respondents attained a high school education (35.42%), followed by those with a diploma (25%), a bachelor’s degree (20.05%), a master’s degree (17.19%), and a minor percentage with a PhD (2.34%).

Regarding age, the largest group of respondents was between 30-40 years old, accounting for 38.54% (148). This was followed by the 17-30 years group (25.78%, 99), the 40-50 years group (21.35%, 82), and smaller proportions in the 50-60 years (9.90%, 38) and 0-17 years (2.08%, 8) groups. Only 2.34% (9) of respondents were above 60 years old.

In terms of occupation, more than half of the respondents were private sector employees (50.78%, 195), while 19.01% (73) were housewives. Entrepreneurs and self-employed individuals represented 9.11% (35) of the sample, and government employees, including those from the army and police, made up 5.21% (20). The remaining 15.89% (61) of respondents fell under the “Other” category, which includes various occupations not specified in the other groups.

### 4.2 Measurement model

The measuring model was evaluated to determine the reliability and validity of the constructs employed in this investigation. The evaluation concentrated on various critical criteria to guarantee the integrity of the measurement model ( Aburumman et al., 2022; Hair et al., 2017, 2019). The criteria encompassed the reliability of the constructs, tested using Cronbach’s Alpha (CA) and Composite Reliability (CR), while convergent validity was examined through indicator loadings and Average Variance Extracted (AVE). Table 3 displays the outcomes of the measurement model evaluation.

**Table 3.** Measurement model assessment.

Variable	Indicators	Loading factor	CA	CR	AVE
Entrepreneurial Leadership	EL1	0.827	0.890	0.912	0.657
	EL2	0.838			
	EL3	0.868			
	EL4	0.887			
	EL5	0.893			
	EL6	0.904			
	EL7	0.905			
	EL8	0.914			
	EL9	0.912			
	EL10	0.886			
	EL11	0.870			
	EL12	0.871			
	EL13	0.871			
Corporate Governance	CG1	0.870	0.915	0.930	0.689
	CG2	0.873			
	CG3	0.896			
	CG4	0.909			
	CG5	0.883			
	CG6	0.887			
	CG7	0.857			
	CG8	0.896			
Service Quality	SQ1	0.899	0.895	0.910	0.710
	SQ2	0.904			
	SQ3	0.897			
	SQ4	0.908			
	SQ5	0.893			
	SQ6	0.901			
	SQ7	0.828			
	SQ8	0.860			
	SQ9	0.830			
Satisfaction	S1	0.892	0.920	0.940	0.749
	S2	0.908			
	S3	0.906			
	S4	0.934			
Patient Loyalty	PL1	0.867	0.895	0.915	0.725
	PL2	0.892			
	PL3	0.863			
	PL4	0.879			
	PL5	0.920			
	PL6	0.893			
	PL7	0.899			
	PL8	0.897			

Source: Author’s processing (2025).

According to Table 3, all items have factor loadings (FL) exceeding 0.7, signifying their validity and appropriateness for assessing their corresponding latent components. The AVE values for all variables surpass the 0.5 threshold, validating that the constructs adequately account for variance in their indicators. Moreover, the findings indicate that all variables and their measurement indicators satisfy the necessary reliability criteria, with both CA and CR ratings surpassing 0.7. The findings indicate that all variables and indicators satisfy the requisite validity and reliability standards, affirming their appropriateness for subsequent study ( Ahmed et al., 2024; Sarstedt et al., 2019).

Following the evaluation of convergent validity and reliability, the subsequent phase in examining the constructs involves the discriminant validity test, executed by the Heterotrait-Monotrait (HTMT) ratio. The HTMT ratio is presented in Table 4.

**Table 4.** HTMT Ratio.

Variable	Entrepreneurial Leadership	Corporate Governance	Service Quality	Satisfaction	Patient Loyalty
Entrepreneurial Leadership	0.763				
Corporate Governance	0.512	0.796			
Service Quality	0.478	0.502	0.781		
Satisfaction	0.532	0.564	0.588	0.838	
Patient Loyalty	0.490	0.529	0.547	0.623	0.746

Source: Author’s processing (2025).

The HTMT analysis findings are displayed in Table 4, which demonstrates that all constructs have HTMT ratio values less than 0.90. This requirement has been satisfactorily satisfied in terms of discriminant validity ( Hair et al., 2017; Henseler et al., 2015). Consequently, every variable satisfies the criteria for discriminant validity and is accepted for the next testing phases.

### 4.3 Structural model

The subsequent phase is to examine the structural model after assessing the reliability and validity of the measurement model. The variance inflation factor (VIF), the coefficient of determination (R ^2^), the cross-validated redundancy measure derived from blindfolding (Q ^2^), and the statistical significance and relevance of path coefficients are among the primary evaluation criteria. The results of the structural model assessment are summarized in Table 5.

**Table 5.** Structural model assesment.

Hypothesis	Path Coefficient	T Statistic	P-values	VIF	f ^2^
H1: Entrepreneurial leadership on corporate governance	0.946	109.029	0.000	1.718	0.172
H2: Entrepreneurial leadership on service quality	0.737	8.088	0.000	1.923	0.216
H3: Corporate governance on patient satisfaction	0.214	2.233	0.026	1.890	0.195
H4: Corporate governance on patient loyalty	0.315	5.389	0.000	2.122	0.255
H5: Service quality on patient satisfaction	0.198	1.337	0.181	2.329	0.278
H6: Service quality on patient loyalty	0.150	2.443	0.015	2.455	0.288
H7: Patient satisfaction on patient loyalty	0.172	2.412	0.016	2.561	0.294

Source: Author’s processing (2025).

The structural model assessment in Table 5 indicates that entrepreneurial leadership exerts a robust and statistically significant influence on corporate governance (path coefficient = 0.946, p-value = 0.000) and service quality (path coefficient = 0.737, p-value = 0.000), underscoring its vital role in improving these elements. Corporate governance has a substantial impact on patient loyalty (path coefficient = 0.315, p-value = 0.000), whereas its effect on patient satisfaction is minimal (path coefficient = 0.214, p-value = 0.026). The correlation between service quality and patient satisfaction is insignificant (path coefficient = 0.198, p-value = 0.181), signifying an absence of direct influence in this model. Service quality strongly influences patient loyalty (path coefficient = 0.150, p-value = 0.015), whereas patient happiness has a favorable effect on patient loyalty (path coefficient = 0.172, p-value = 0.016). Entrepreneurial leadership profoundly affects governance and service quality, thus influencing patient loyalty, while patient satisfaction is equally crucial in cultivating loyalty.

Moreover, the VIF values, all beneath the threshold of 5, signify the absence of multicollinearity issues inside the model. This guarantees the stability and reliability of the path coefficient estimations, affirming that the relationships among the variables are not skewed by high correlation among predictors. The effect size (f ^2^) values demonstrate moderate to substantial impacts for the principal relationships in the model. Corporate governance (f ^2^ = 0.255), service quality (f ^2^ = 0.288), and patient satisfaction (f ^2^ = 0.294) have significant effects, with the most pronounced influence noted for patient satisfaction on patient loyalty (f ^2^ = 0.294). The R ^2^ values presented in Table 6 for corporate governance, service quality, patient happiness, and patient loyalty are 0.895, 0.908, 0.822, and 0.949, respectively. The elevated R ^2^ values indicate that the model accounts for a significant percentage of the variance in each construct, signifying a strong fit between the model and the data. The Q ^2^ values exceed 0.5 for all constructs, signifying that the model possesses substantial predictive relevance.

**Table 6.** R-square and Q-square value.

Construct	R ^2^	Q ^2^
Corporate Governance	0.895	0.891
Service Quality	0.908	0.902
Patient Satisfaction	0.822	0.817
Patient Loyalty	0.949	0.935

Source: Author’s processing (2025).

## 5. Discussion

This study’s findings offer significant insights into the interconnections among entrepreneurial leadership, corporate governance, service quality, patient satisfaction, and patient loyalty within healthcare environments. The examination of the structural model indicates the crucial influence of entrepreneurial leadership on improving corporate governance and service quality, along with its indirect impact on patient happiness and loyalty.

Entrepreneurial leadership demonstrates a strong positive influence on corporate governance, as evidenced by the high path coefficient and significant p-value. This finding highlights the importance of proactive, innovative, and risk-taking leadership in shaping governance structures within healthcare organizations. Leaders who adopt an entrepreneurial mindset foster transparency, accountability, and ethical decision-making, which are essential for ensuring effective governance. In healthcare settings, especially those in rapidly changing environments, entrepreneurial leadership helps organizations remain adaptable, competitive, and patient-centered. By adopting innovative governance structures, these leaders can drive operational efficiency, enhance patient care, and maintain high standards of ethical practice. This aligns with previous research indicating that entrepreneurial leadership is key to the sustainable success of organizations ( Clarks et al., 2019; Kebede et al., 2024).

Similarly, the significant relationship between entrepreneurial leadership and service quality underscores the critical role of leadership in improving the quality of care provided. Entrepreneurial leaders prioritize continuous improvement, innovation, and customer-centric strategies, which directly contribute to the enhancement of service quality. These leaders encourage staff creativity, empower teams to innovate, and foster a culture of responsiveness, all of which lead to improved patient outcomes and satisfaction. The path coefficient further supports the idea that entrepreneurial leadership is a significant driver of service quality in healthcare organizations ( Renko et al., 2015; Kim et al., 2018). Healthcare institutions led by entrepreneurial figures are better equipped to incorporate new technologies, respond to patient needs, and continuously refine service delivery to meet evolving expectations.

However, while corporate governance was found to significantly influence both patient satisfaction and patient loyalty, the effect on patient satisfaction is moderate. This indicates that although strong governance practices contribute to improving patient satisfaction by ensuring transparency, ethical decision-making, and efficient resource allocation, other factors, such as service quality and leadership, may play more substantial roles. In healthcare, patient satisfaction is highly influenced by the direct interactions patients have with healthcare providers, as well as the overall service environment. Governance, while essential for creating an ethical and patient-centered environment, may not be as directly impactful on satisfaction compared to more immediate service experiences.

In contrast, corporate governance has a more pronounced effect on patient loyalty, indicating that patients are more likely to remain loyal to providers that exhibit strong ethical standards, transparency, and accountability. Effective governance practices help establish long-term trust between patients and healthcare organizations, which is a crucial factor in patient loyalty. Hospitals with robust governance structures are more likely to consistently deliver high-quality care, address patient concerns in a timely manner, and adapt to changes in patient needs. These factors contribute to sustained patient loyalty, as patients perceive these organizations as reliable and trustworthy ( Meesala & Paul, 2018).

The lack of a significant relationship between patient satisfaction and service quality implies that, in this model, patient satisfaction may not be directly and substantially influenced by service quality alone. This discovery may suggest that, although service quality is crucial, satisfaction levels are more significantly influenced by other variables, including interpersonal communication, emotional engagement, and patient-provider relationship. There is a possibility that patient satisfaction is more significantly impacted by factors such as staff empathy, communication, and personal care in healthcare settings than by the technical quality of the services provided.

Conversely, it was found that patient loyalty is substantially influenced by service quality, which confirms that patients who perceive high service quality are more inclined to remain loyal to their healthcare provider. This is especially crucial in a healthcare market that is highly competitive, as patients have a plethora of choices. Healthcare providers that provide high-quality services, which are defined by responsiveness, empathy, and reliability, are more likely to establish strong emotional connections with their patients. These connections are essential for cultivating long-term loyalty. This is consistent with the Social Exchange Theory, which posits that patients perceive value from their healthcare provider, resulting in increased engagement and loyalty ( Naidu, 2009; Swain & Kar, 2018).

Furthermore, the substantial influence of patient satisfaction on patient loyalty underscores the necessity of addressing both the technical and affective components of care. The likelihood of patients remaining loyal to their healthcare provider is higher when they are satisfied with the care they receive, including emotional support and clinical outcomes. The relational aspects of patient care, including the extent to which patients feel heard, respected, and comprehended, are equally important as clinical outcomes in the development of enduring loyalty ( Dayan et al., 2022; Liu et al., 2021).

## 6. Conclusion

The data analysis reveals that entrepreneurial leadership has a significant positive effect on both corporate governance and service quality. Entrepreneurial leaders, by fostering innovation and proactivity, enhance governance structures that promote transparency, accountability, and ethical decision-making, leading to improved service delivery. Corporate governance was found to have a positive impact on both patient satisfaction and patient loyalty, with well-structured governance contributing to higher satisfaction and long-term loyalty. However, while corporate governance influences patient satisfaction, its effect is moderate compared to service quality and leadership. Service quality, although not directly influencing patient satisfaction, was found to significantly affect patient loyalty, showing that high service quality fosters emotional connections and long-term loyalty. Lastly, patient satisfaction was confirmed to be a critical driver of patient loyalty, as satisfied patients are more likely to return and recommend the healthcare provider.

From a practical standpoint, healthcare organizations must focus on fostering entrepreneurial leadership, improving corporate governance, and prioritizing service quality to enhance patient satisfaction and loyalty. Leaders should be equipped with the skills to drive innovation, manage risks, and adapt to changing patient expectations. Furthermore, healthcare managers should ensure that governance structures are ethical, transparent, and accountable, with a focus on patient-centered care. Investment in service quality, particularly through staff training, process optimization, and patient feedback mechanisms, is also essential for creating a positive patient experience.

This research has significant implications for the practice of healthcare. It implies that healthcare organizations should allocate resources to leadership development programs in order to cultivate entrepreneurial leadership that can facilitate patient care and innovation. In turn, healthcare providers will be able to operate with greater transparency and accountability, which will increase patient satisfaction and loyalty, as a result of the strengthening of corporate governance policies. Lastly, healthcare organizations will be able to maintain their competitiveness and establish enduring relationships with patients by continuing to prioritize service quality enhancements through staff training, patient engagement, and technological advancements. In the future, longitudinal studies should be conducted to evaluate the long-term impact of leadership, governance, and service quality on patient loyalty, as well as the potential mediation effects between the variables. The generalizability of the results could be improved by extending the findings to other healthcare systems, as this research is also founded on a specific sample.

## Ethical approval

This study involving human participants was conducted in accordance with the ethical standards of the institutional research committee and with the 1964 Helsinki Declaration and its later amendments or comparable ethical standards. Ethical approval for all study procedures was obtained from the Ethics Committee of the Faculty of Administrative Science, University of Brawijaya, with approval number: 06210/UN10.F0301/B/PP/2025.

Prior to participation, informed consent was obtained verbally from all respondents. Verbal consent was chosen over written informed consent due to the nature of the study, which involved minimal risk and was conducted in informal, community-based settings where participants expressed hesitation in signing formal documents. Many participants were more comfortable providing verbal agreement, which was considered culturally appropriate and less intimidating. The procedure for obtaining verbal consent, including a clear explanation of the study’s purpose, risks, benefits, and confidentiality measures, was approved by the Ethics Committee. The anonymity and confidentiality of participants were strictly maintained, and no personally identifiable information was collected. Participation in the study was entirely voluntary and did not influence any rights or access to services of the participants.

## Data Availability

Raw Data for “Exploring Patient Loyalty: The Effect of Entrepreneurial Leadership Through Corporate Governance, Service Quality, and Satisfaction”, DOI: dx.doi.org/10.6084/m9.figshare.29625374 ( Hanafi et al., 2025c). Figshare: Questionnaire for “Exploring Patient Loyalty: The Effect of Entrepreneurial Leadership Through Corporate Governance, Service Quality, and Satisfaction”, DOI: dx.doi.org/10.6084/m9.figshare.29486258 ( Hanafi et al., 2025a). STROBE checklist for “Exploring Patient Loyalty: The Effect of Entrepreneurial Leadership Through Corporate Governance, Service Quality, and Satisfaction”, DOI: dx.doi.org/10.6084/m9.figshare.29486288 ( Hanafi et al., 2025b). Data are available under the terms of the Creative Commons Attribution 4.0 International license (CC-BY 4.0).
