# Supplementary material for: Entrepreneurial Leadership and Patient Loyalty in Indonesian Hospitals: The Roles of Corporate Governance, Service Quality, and Patient Satisfaction
Source: F1000Res. 2025 Nov 14;14:1040. Originally published 2025 Oct 3. [Version 2] doi: 10.12688/f1000research.166837.2 (PMC12640491; doi:10.12688/f1000research.166837.2)
Supplement: Peer review history

# Reviewer report: Reviewer response for version 2

**DOI:** 10.5256/f1000research.191071.r432540

**Published:** 2025-11-22

**Authors:** Anis Ansyori

**Affiliations:** 1 Institute of Science and Health Technology, Dr. Soepraoen Hospital, Malang, Indonesia

After reviewing the authors' revisions and their responses to my previous comments, I believe all issues have been addressed well. The revisions have clarified and strengthened the article overall. The authors have provided clear and appropriate responses to the feedback. I have no further comments and fully support the publication of this article.

Is the work clearly and accurately presented and does it cite the current literature?

Yes

If applicable, is the statistical analysis and its interpretation appropriate?

Yes

Are all the source data underlying the results available to ensure full reproducibility?

Yes

Is the study design appropriate and is the work technically sound?

Yes

Are the conclusions drawn adequately supported by the results?

Yes

Are sufficient details of methods and analysis provided to allow replication by others?

Yes

Reviewer Expertise:

Public relations and strategic health communication, health literacy

I confirm that I have read this submission and believe that I have an appropriate level of expertise to confirm that it is of an acceptable scientific standard.

# Reviewer report: Reviewer response for version 2

**DOI:** 10.5256/f1000research.191071.r432539

**Published:** 2025-11-22

**Authors:** Mohammed Sallam

**Affiliations:** 1 Mediclinic Parkview Hospital, Mediclinic Middle East, Dubai, United Arab Emirates

Thank you for the thoughtful and well-executed revisions; the clarifications and improvements effectively address the earlier concerns and strengthen the manuscript.

Is the work clearly and accurately presented and does it cite the current literature?

Partly

If applicable, is the statistical analysis and its interpretation appropriate?

I cannot comment. A qualified statistician is required.

Are all the source data underlying the results available to ensure full reproducibility?

Yes

Is the study design appropriate and is the work technically sound?

Yes

Are the conclusions drawn adequately supported by the results?

Yes

Are sufficient details of methods and analysis provided to allow replication by others?

Yes

Reviewer Expertise:

Drug Information, Hospital Pharmacy, Pharmacovigilance, Pharmacy Education, Pharmacodynamics, Patient Safety, SOP, Clinical Data Management, Descriptive Studies, Pharmacy Management, Medical Societies, Transformational Leadership, Lean Six Sigma, Lean Management, Operations Management, Total Quality Management, Drug Utilization Review, Antimicrobial Stewardship, Antimicrobial Resistance, Medication Safety, Team Management, Pharmacy Administration, DMAIC, Artificial Intelligence, Action Research, Process Improvement, Logistics Management, Time-Driven Activity-Based Costing (TDABC) in Healthcare, Obesity Management, Pharmacoeconomics

I confirm that I have read this submission and believe that I have an appropriate level of expertise to confirm that it is of an acceptable scientific standard.

# Reviewer report: Reviewer response for version 1

**DOI:** 10.5256/f1000research.183885.r424374

**Published:** 2025-10-25

**Authors:** Anis Ansyori

**Affiliations:** 1 Institute of Science and Health Technology, Dr. Soepraoen Hospital, Malang, Indonesia

What the paper does well:

- Important topic for hospitals: how leadership and governance drive service quality, satisfaction, and loyalty.
- Strong method: survey of 384 “executive” patients in West Java; PLS-SEM with solid reliability/validity.
- Clear findings: entrepreneurial leadership strongly improves governance and service quality; governance, service quality, and satisfaction support patient loyalty.
- Useful, practical implications for managers and policymakers.

Small fixes before acceptance:

1. Make results consistent: The abstract says service quality affects satisfaction, but the model shows this path is not significant—please align wording across abstract/results/discussion.
2. Clarify “executive patients” **:** Define the segment briefly and note limits for generalization.
3. Complete tables/figures: Ensure all tables (samples, demographics, measurement, structural paths) and figures (trend chart, model with coefficients/p-values) are fully labeled and readable.
4. Sampling/math details **:** Show the proportional allocation per hospital and the Slovin formula clearly (with e=0.05).
5. Minor editing **:** Fix typos/name inconsistencies and standardize references/citation style.

Bottom line:

 A well-designed and relevant study. With these minor clarifications and formatting tweaks, it’s ready for indexing.

Is the work clearly and accurately presented and does it cite the current literature?

Yes

If applicable, is the statistical analysis and its interpretation appropriate?

Yes

Are all the source data underlying the results available to ensure full reproducibility?

Yes

Is the study design appropriate and is the work technically sound?

Yes

Are the conclusions drawn adequately supported by the results?

Yes

Are sufficient details of methods and analysis provided to allow replication by others?

Yes

Reviewer Expertise:

Public relations and strategic health communication, health literacy

I confirm that I have read this submission and believe that I have an appropriate level of expertise to confirm that it is of an acceptable scientific standard, however I have significant reservations, as outlined above.

# Reviewer report: Reviewer response for version 1

**DOI:** 10.5256/f1000research.183885.r421621

**Published:** 2025-10-14

**Authors:** Mohammed Sallam

**Affiliations:** 1 Mediclinic Parkview Hospital, Mediclinic Middle East, Dubai, United Arab Emirates

Title:

 - Please revise the title to improve readability, specify the study context (Indonesian hospitals), and clearly reflect the mediating relationships. Suggestion: “Entrepreneurial Leadership and Patient Loyalty in Indonesian Hospitals: The Mediating Roles of Corporate Governance, Service Quality, and Patient Satisfaction”

 Abstract:

 - Please revise the phrase “This investigation investigates” to “This study examines the relationships...”

 - Please simplify the opening sentence for clarity and impact.

 - Please replace “This investigation investigates” with “This study examines.”

 - Please mention the study context (Indonesia) in the background to orient readers early.

 - Please highlight the specific research gap “Few studies have empirically examined how entrepreneurial leadership and governance jointly shape patient satisfaction and loyalty in Indonesian hospitals”

 - Please add the study design “A cross-sectional survey was conducted…”

 - Please clarify why “executive patients” were chosen and how they represent the population

 - Please briefly mention the validated measurement sources or constructs

 - Please include key statistical indicators p-values

 - Please clarify the unexpected finding “service quality moderately influencing satisfaction”

 - Please specify the theoretical implication more clearly

 - Please conclude with a forward-looking statement highlighting the study’s broader implications

 Introduction:

 - The Introduction demonstrates comprehensive background knowledge and strong contextual grounding

 - Please focus on logical sequencing 1. Problem, 2. Gap, 3. Objective

 - Please integrate theory-based relationships

 - Please refine transitions to ensure coherence between entrepreneurial leadership, governance, service quality, and satisfaction leading to loyalty

 Literature review and hypothesis development:

 - Please operationalize governance for hospitals and define governance dimensions you are using

 - Please define “sustainability”

 - H1 is directional but underspecified; “effect” could be positive or negative; also, level-of-analysis unclear. Please specify positive direction and level (organizational)

 - H2 direction missing; mediation intent not prefigured. Please state positive effect and indicate whether service quality mediates entrepreneurial leadership

 - H3: Please specify positive effect and please predefine level

 - H4: Please state whether you expect a direct positive effect and please add a mediation hypothesis

 - H5: Please state expected positive sign clearly

 - H6: Please add a mediation hypothesis and pre-specify testing

 - H7: Please add full-path mediation hypotheses and preregister which direct effects you expect to remain significant when mediators are included

 Research methods:

 - Please define “executive patient” operationally and please justify why this subgroup represents loyalty dynamics

 - Please specify the hospital sampling method (random or stratified) and compare selected vs non-selected hospitals on size, ownership, location to support representativeness

 - Please justify the ≥2 visits rule

 - Please justify how patients can reliably assess governance

 - Please justify PLS choice and report power analysis for the most complex regression

 - ““The data were analyzed using Partial Least Squares (PLS) via SmartPLS 4.0. PLS is a robust technique that does not require the data to conform to the normality assumptions…”

 Replace “resilient” with “robust” and “normalcy” with “normality”

 Results:

 - Please verify the appropriateness of including minors (0–17 years) in a survey targeting “executive patients

 - Please explain why male respondents dominate the sample (74.7%)? whether due to hospital policy, socioeconomic patterns, or sampling bias

 - Please discuss practical implications of key significant paths

 Discussion:

 - Please restate the purpose and confirm which hypotheses were supported or not supported

 - Please elaborate how entrepreneurial leadership behaviors operationally enhance governance outcomes

 - Please add the following supporting sentence to strengthen the discussion with a recent, real-world example that reinforces the empirical validity of your study. The cited study provides contemporary evidence linking leadership style and structured quality frameworks (Lean Six Sigma) to measurable improvements in healthcare service outcomes.

 “Similarly, the significant relationship between entrepreneurial leadership and service quality underscores the critical role of leadership in improving the quality of care provided. Recent findings from a Lean Six Sigma–driven improvement initiative at Mediclinic Parkview Hospital in Dubai highlighted how leadership style that integrates innovation with structured governance frameworks can substantially elevate healthcare service quality, patient satisfaction, and operational performance ( https://pharmacypractice.org/index.php/pp/article/view/3263)”

 - Please differentiate between cognitive trust (governance-based) and affective trust (experience-based)

 - Please add a clear Study Limitations subsection after the Discussion

 1. Please acknowledge the cross-sectional design, which prevents causal inference.

 2. Please note that the data were self-reported from executive patients, which may introduce selection or response bias and limit generalizability.

 3. Please mention the geographical limitation to Indonesian hospitals, suggesting caution in applying findings to other healthcare systems.

 4. Please indicate that PLS-SEM analysis assumes linear relationships and does not capture complex nonlinear interactions.

 Conclusion:

 - Please emphasize theoretical contribution rather than restating statistical effects (highlight how entrepreneurial leadership operationally reinforces governance and service excellence in healthcare). Emphasize novelty in linking entrepreneurial leadership and governance to loyalty

 - Please avoid causal language; use “is associated with” or “predicts” considering the cross-sectional study design

 General:

 - Please ensure terminological and conceptual consistency. Use “patient satisfaction,” not “happiness”

 - Please include the full questionnaire as an appendix/supplementary file

 - Please review all figures and tables for clarity and labelling

 ‎- Please ensure that the reference list follows journal formatting guidelines

 ‎- Please ensure English proofreading is completed to maintain grammatical consistency, ‎avoid spelling mistakes, tense consistency, and ensure a uniform concise academic tone ‎throughout the manuscript

Is the work clearly and accurately presented and does it cite the current literature?

Partly

If applicable, is the statistical analysis and its interpretation appropriate?

I cannot comment. A qualified statistician is required.

Are all the source data underlying the results available to ensure full reproducibility?

Yes

Is the study design appropriate and is the work technically sound?

Yes

Are the conclusions drawn adequately supported by the results?

Yes

Are sufficient details of methods and analysis provided to allow replication by others?

Yes

Reviewer Expertise:

Drug InformationHospital PharmacyPharmacovigilancePharmacy EducationPharmacodynamicsPatient SafetySOPClinical Data ManagementDescriptive StudiesPharmacy ManagementMedical SocietiesTransformational LeadershipLean Six SigmaLean ManagementOperations ManagementTotal Quality ManagementDrug Utilization ReviewAntimicrobial StewardshipAntimicrobial ResistanceMedication SafetyTeam ManagementPharmacy AdministrationDMAICArtificial IntelligenceAction ResearchProcess ImprovementLogistics ManagementTime-Driven Activity-Based Costing (TDABC) in HealthcareObesity managementPharmacoeconomics

I confirm that I have read this submission and believe that I have an appropriate level of expertise to confirm that it is of an acceptable scientific standard, however I have significant reservations, as outlined above.
